# Chemical Composition and Larvicidal Activity against *Aedes aegypti *Larvae of Essential Oils from Four *Guarea *Species 

**DOI:** 10.3390/molecules15085734

**Published:** 2010-08-19

**Authors:** Lyege Amazonas Maciel Magalhães, Maria da Paz Lima, Marcia Ortiz Mayo Marques, Roselaine Facanali, Ana Cristina da Silva Pinto, Wanderli Pedro Tadei

**Affiliations:** 1 Coordenação de Pesquisas em Produtos Naturais, Instituto Nacional de Pesquisas da Amazônia, CP 478, 69011-970 Manaus-AM, Brazil; E-Mail: lyegemagalhaes@yahoo.com.br (L.A.); 2 Instituto Agronômico de Campinas, CP 28, 13001-970, Campinas, SP, Brazil; E-Mails: mortiz@iac.sp.gov.br (M.O.); rosefacanali@ig.com.br (R.F.); 3 Laboratório de Vetores de Malária e Dengue, Instituto Nacional de Pesquisas da Amazônia, CP 478, 69011-970 Manaus-AM, Brazil; E-Mails: anacristinadsp@gmail.com (A.C.); wptadei@gmail.com (W.P.)

**Keywords:** *Guarea convergens*, *Guarea humaitensis*, *Guarea scabra*, *Guarea silvatica*, Meliaceae

## Abstract

The essential oils of four *Guarea* species collected at Manaus (Amazonas, Brazil) were obtained by hydrodistillation and analyzed by GC-MS. Except for one diterpene detected, the compounds identified in the essential oils were hydrocarbons and oxygenated sesquiterpenes. The major sesquiterpenes were α-santalene (26.26%) and α-copaene (14.61%) from *G. convergens* branches; caryophyllene epoxide (40.91%) and humulene epoxide II (14.43%) from *G. humaitensis* branches; *cis*-caryophyllene (33.37%) and α-*trans*-bergamotene (11.88%) from *G. scabra* leaves; caryophyllene epoxide (36.54%) in leaves and spathulenol (14.34%) in branches from *G. silvatica*. The diterpene kaurene (15.61%) was found in *G. silvatica* leaves. Larvicidal activity assay of essential oils against third-instar *Aedes aegypti *larvae revealed that at higher concentrations (500 and 250 μg/mL), all the essential oils caused 100% mortality after 24 h of exposure. The most active essential oils were those of *G*. *humaitensis* branches (LC_50_ 48.6 μg/mL), *G. scabra* leaves (LC_50_ 98.6 μg/mL) and *G. silvatica* (LC_50_ 117.9 μg/mL). The differences in the toxicity of essential oils of *Guarea* species on *A. aegypti* are due to qualitative and quantitative variations of the components, therefore the larvicidal effect may be due to higher amount of the sesquiterpenes with caryophyllane skeleton.

## 1. Introduction

Dengue is a viral disease that has major public health consequences in many parts of the world. The principal vector of dengue fever, including the haemorrhagic form, is the mosquito *Aedes aegypti * L. (Diptera, Culicidae) [1]. Presently, mosquito control primarily depends worldwide on continued applications of conventional toxic synthetic insecticides to which resistance has been reported in many areas where it is widely used [2,3,4]. This has stimulated the investigation of natural insecticides as an alternative control, focused on plant-derived compounds, including volatile chemical constituents (essential oils), as potentially bioactive substances against mosquito larvae. 

In nature, many essential oils play an important role in protecting plants against pathogens and also against herbivores by reducing their appetite for such plants. They also may attract some insects to favour the dispersion of pollens and seeds, or act as repellent for other undesirable insects [5]. In addition, some of them have been shown to be effective in the control of mosquito larvae responsible for the transmission of dengue fever. Essential oils with larvicidal activity against third-instar of *A. aegypti *have been extracted from plants of the Myrtaceae [6], Piperaceae [7], Poaceae [8], Lamiaceae [8,9,10], Rutaceae [8,9,11,12], Verbenaceae [8,9], Apiaceae and Zingiberaceae [11]. There are however no published reports on larvicidal activity of Meliaceae essential oils. In this study we analyzed the constituents of essential oils from species of this family *Guarea convergens, G. humaitensis, G. scabra, G. silvatica* and studied their larvicidal activity against *A*. *aegypti*.

## 2. Results and Discussion

### 2.1. Yields and chemical analysis

The yields of essential oils obtained from *Guarea* species via hydrodistillation ranged from 0.1% to 1.3% (based on the dry weight of the plant material). Except for one diterpene detected, the compounds identified from the five essential oils (Table 1) were hydrocarbons and oxygenated sesquiterpenes. The identified terpenoids, together with their Kovats index (KI) and the percentages of essential oils are shown in Table 2. Sixteen sesquiterpenes were identified in *G. convergens* branches, with predominance of sesquipinane (α-santalene; 26.26%) and copaane (α-copaene; 14.61%) skeletons. Among the thirteen sesquiterpenes from *G. humaitensis* branches, those with caryophyllane skeletons (caryophyllene epoxide; 40.91%) and humulane (humulene epoxide II; 14.43%) were predominant. In *G. scabra *leaves a high percentage of sesquiterpene caryophyllane-type (*cis*-caryophyllene; 33.37%) and elemene-type (α-*trans*-bergamotene; 11.88%) was also found. The diterpene kaurene (15.61%) was found in essential oil *G. silvatica* leaves, but the smost abundant esquiterpene was caryophyllene epoxide (36.54%). In the essential oil of branches of this species, the oxygenated sesquiterpenes spathulenol (14.34%) and caryophyllene epoxide (8.57%) were identified as major constituents. 

Despite the fact that the genus *Guarea* includes more than 50 species, few studies on the essential oils of its species have been published, namely, the Brazilian species *G. guidonia* [13,14,15], *G. macrophylla ssp tuberculata* [16,17,18] and the African species *G. cedrata* [19], whose dominant compounds differ from those found in the present study.

**Table 1:** Yields of essential oils and types of terpenoids identified.

Essential oil	Yields (%)	No. of Constituents	Sesquiterpene		Diterpene
			H	O	
GCb	0.1	16	13	3	
GHb	0.3	13	10	3	
GSl	0.2	17	14	3	
GSil	1.3	9	5	3	1
GSib	0.1	18	11	7	

O-oxygenated; H-hydrocarbons; GCb, *Guarea convergens * branches; GHb, *Guarea humaitensis* branches; GSl, *Guarea scabra* leaves; GSil, *Guarea silvatica* leaves; GSib, *Guarea silvatica* branches

**Table 2:** Chemical compositions of essential oils from *G. convergens*, *G. humaitensis*, *G. scabra* and *G. silvatica.*

Compounds	KI*	Relative content ( %)				
		GCb	GHb	GSl	GSil	GSib
δ-Elemene	1336		1.01	2.60		
α-Ylangene	1370			0.71		
α-Copaene	1375	14.61	1.84	2.44	2.81	
β-Bourbonene	1383			5.42	0.40	
β-Elemene	1390	1.95	2.27	0.88	6.20	
*cis*-Caryophyllene	1405			33.37		
α-*cis*-Bergamoteme	1414			1.28		
α-Santalene	1419	26.26				
β-Gurjunene	1427			2.08		
α-*trans*-Bergamotene	1436	2.32	1.07	11.88		0.73
α-Humulene	1450			1.02		
β-Santalene	1457	6.32	0.98			
Drima-7,9(11)-diene	1467	6.85				
γ-Muurolene	1475		3.71	5.00	1.05	2.91
Ar-curcumene	1479	2.02	2.05	2.67		
β-Selinene	1485	1.88			1.06	0.69
α-Selinene	1492	2.02				
*cis*-Cadina-1,4-diene	1493					0.86
α-Muurolene	1498	1.72		2.36		1.65
β-Bisabolene	1508	5.18	8.36			2.90
γ-Cadinene	1512		1.97	3.48		2.15
6-Methyl-α-ionone	1517					0.87
*cis*-Calamenene	1520	2.61				1.10
(*E*)-*iso*-γ-Bisabolene	1525					2.31
Spathulenol	1576	3.99			4.17	14.34
Caryophyllene epoxide	1579	1.27	40.91	0.54	36.54	8.57
*trans*-Nerolidol	1560			1.63		
Humulene epoxide II	1605		14.43		2.12	239
1-*epi*-Cubenol	1625	1.77	2.60			1.90
*epi*-α-Cadinol	1639			3.30		3.27
β-Eudesmol	1646					4.65
Cadalene	1670	1.27	0.94			2.85
Mustakone	1672					1.33
Kaurene	2034				15.61	

Sesquiterpenes (%) no identified in GSIg: IK 1532 (1.05), 1548 (0.96), 1584 (0.95), 1588 (2.27), 1592 (3.76), 1643 (3.17), 1650 (4.68), 1654 (1.54), 1699 (2.68), 1712 (1.24), 1739 (1.27), 1749 (1.28), 1761 (4.03), 1763 (2.12), 1860 (5.47), 1967 (6.94), 2109 (1.14).

### 2.2. Larvicidal activity of essential oils

The five essential oils obtained were investigated for their larvicidal activities against third-instar *A. aegypti* larvae. At higher concentrations (500 and 250 μg/mL), all the essential oils exhibited 100% mortality after 24 h of exposure; the most active were those *Guarea humaitensis* branches (LC_50_ 48.6 μg/mL), *G. scabra* leaves (LC_50_ 98.6 μg/mL) and *G. silvatica *leaves (LC_50_ 117.9 μg/mL), in accordance with Table 3. The differences in the toxicity of essential oils of *Guarea* species against *A. aegypti* were due to both qualitative and quantitative variations of the components, therefore the larvicidal effect may be due to higher amount of sesquiterpenes with caryophyllane skeletons: caryophyllene epoxide (40.91%, GHb and 36.54%, GSil), and *cis*-caryophyllene (33.37%, GSl). 

**Table 3:** LC_50_ and LC_90 _values for 24 h with their 95% confidence limits, standard deviation, slope and Chi-square(χ^2^) of essential oils against third larvae *A. aegypti. *

Oils	LC_50 _(CL)	LC_90 _(CL)	sd	Slope	χ^2^ (df)
*G. convergens* branches	145.1 (133.2 –158.8)	218.2 (195.7 – 252.2)	1.5	7.2 ± 0.7	0.04** (3)
*G. humaitensis* branches	48.6 (37.9 – 64.0)	80.2 (61.7 – 158.1)	1.0	5.9 ± 0.6	8.69* (3)
*G. scabra* leaves	98.6 (90.8 – 108.1)	158.8 (139.8 – 191.4)	1.4	6.2 ± 0.7	1.12** (3)
*G. silvatica* leaves	117.8	261.7	0.6	3.7 ± 0.3	89.77* (3)
*G. silvatica* branches	273.6	424.3	1.9	6.7 ± 0.8	30.49* (3)

LC_50_ = Lethal concentration (μg/mL) at which 50% of the larvae showed mortality. LC_90_ = Lethal concentration (μg/mL) at which 90% of the larvae showed mortality. * significative; ** not significative.

Several studies have shown that sesquiterpenoids possess significant larvicidal activity against *A. aegypti. *Cheng *et al*. [20] reported the results of screening essential oils and suggested that oils presenting LC_50_ values > 100 ppm should not be considered active, whereas those with LC_50_ values < 50 ppm could be regarded as highly active. The test using known compounds conducted by Cheng *et al*., [21] in fouth-instar mosquito larvae *A. albopictus* included caryophyllene epoxide, which displayed a LC_50_ value of 65.6 μg/mL at 24 h of exposure.

## 3. Experimental

### 3.1. Plant material and oil distillation

The samples of *G. convergens*, *G. humaitensis, G. scabra* and *G. silvatica* were collected in November, 2006 at the Forest Reserve Adolfo Ducke, Km 26 from Manaus, Amazonas, Brazil from individual previously marked (trees no. 4451-09, 4705-09, 3095-09 and 2091-09, respectively) and identified by Dr. Terence D. Pennington during the “Flora da Reserva Ducke” project Instituto Nacional de Pesquisas da Amazônia (INPA) [22]. The leaves and branches were dried in an air conditioned room at 25 °C for seven days, milled and submitted to hydrodistillation in a Clevenger-type apparatus for 4 hours. The oils were dried in anhydrous sodium sulphate, filtered, stored in amber glass bottles in a refrigerator (4 °C) for investigation of chemical constituents and larvicidal activity. 

### 3.2. Gas chromatography-mass spectrometry (GC/MS)

Analyses of volatile constituents were performed on a Shimadzu QP5000 instrument, equipped with a DB-5 fused silica capillary column (J&W Scientific – Serial N^0^ 8766726; 5% phenylmethylsiloxane; 30 m × 0.25 mm × 0.25 μm). The electron impact technique (70 eV) was used with the injector temperature at 240 °C and the detector at 230 °C. The carrier gas was helium at the working rate of 1.0 mL/min. The column temperature was initially 60°C and then was gradually increased at the rate of 3 °C/min up to 240 °C. Compounds were identified by comparing their mass spectrum to those of the database of the GC-MS (NIST 62.lib), literature [23] and retention indices [24]. 

### 3.3. Larvicidal bioassay

Larvae of *Aedes aegypti* were obtained from a permanent colony, maintained at temperature of 23–27 °C and relative humidity of 50–70% in the Laboratório de Vetores de Malária e Dengue of the Instituto Nacional de Pesquisas da Amazônia. The essential oils were dissolved in DMSO (20 mg/mL) and aliquots of the stock solution in appropriate amounts for different concentrations (500, 250, 100, 50 and 25 μg/mL) to the final volume of 5 mL were transferred to plastic cups containing distilled water and food. Then, thirty third-instar larvae of *A. aegypti* were placed in each cup. After 24 hours, the number of dead larvae was counted and the lethal percentage calculated. Each experiment was performed in triplicate and a control test was run in parallel using 5 mL of distilled water and 125 μL of DMSO solution. 

### 3.4. Statistical analysis

Data were evaluated through Probit analysis (POLO PC program) to determine the LC_50_ and LC_90_, representing the concentrations in μg/mL that caused 50 and 90% mortality along with 95% confidence intervals [25]. The 95% confidence intervals, values and degrees of freedom of the χ^2^ goodness of fit tests, and regression equations were recorded. Essential oils from *G. silvatica* (leaves and branches) does not constitute a confidence interval because the data does not apply to the Probit model, because the value of χ^2 ^calc. > χ^2^ tab (7.8, P = 0.05 and three degrees of freedom). That is, determining the value estimate of the confidence interval is calculated when it is ≤ 0.5 which did not occur in the case of these oils.

## 4. Conclusions

This is the first report on the composition and larvicidal activity against *Aedes aegypti* from essential oils of *Guarea convergens*, *G. humaitensis*, *G. scabra* and *G. silvatica*. The results obtained from the larvicidal test indicated that the essential oils containing sesquiterpenes with caryophyllane skeletons as major compounds were the most active, suggesting that caryophyllene epoxide and *cis*-caryophyllene are probably the active principles responsible for the observed *A. aegypti* larvicidal action, therefore further studies for the mode of the actions these constituents are necessary. These results can be used in the research for natural biodegradable larvicidal, compounds, besides contributing for to the knowledge of the aromatic flora of the Biological Preserves of the Amazonian forest. 
